# Recurrent inflammatory myofibroblastic tumors (IMTs) of bladder managed with transurethral resection; case report

**DOI:** 10.1016/j.ijscr.2025.110978

**Published:** 2025-01-29

**Authors:** Abiy Tadele Alene, Kumlachew Tilahun Tamir, Admassu Melaku, Melat Debebe Teka, Endale Demeke Desalew

**Affiliations:** Addis Ababa University, College of Health Sciences, School of Medicine, Ethiopia

**Keywords:** Case report, Recurrent inflammatory myofibroblastic tumor of bladder, Transuretheral resection of bladder tumor

## Abstract

**Introduction and importance:**

Bladder IMTs are uncommon subtypes of sarcoma arising from mucosal epithelium and mesentery. They can occur various organs like eyes, nose, mouth, digestive tract, lungs, and genital and urinary tracts [1]. Typically benign, these tumors can occasionally exhibit local aggression. Diagnosis challenging due to its pathology confusion with bladder sarcomas, immunohistochemical analysis may be necessary to differentiate [6].

Here we report a 52 years old lady with recurrent bladder IMT who was treated with transurethral resection of bladder tumor(TURBT). This case report is in line with criteria from updated c Surgical CAse REport (SCARE) guidelines. [11]Importance of this report is help us to understand nature, prognosis and management of this rare tumor.

**Case presentation:**

A 52 years old lady presented from Silte,Ethiopia with gross painless hematuria of 2 weeks duration associated storage Lower urinary tract symptoms. She had TURBT done 8 months back for this she is on follow up at Outpatient department.

**Clinical discussion:**

Inflammatory myofibroblastic tumor (IMT) is a very rare mesenchymal neoplasm that tends to occur in children and young adults, with a mean age of 9 to 10 years.

Because of its highly cellular nature and aggressive behavior, it can be confused with malignancy. Pathologic evaluation and full histopathological analysis is recommended for establishing diagnosis. Clinical presentation mostly dependant on site of origin in case of bladder IMT gross hematuria most common presentation, supra pubic pain, storage lower urinary tract symptoms and if bleeding is severe enough they may have systemic symptoms of anemia.Management is aimed at complete local resection through transurethral resection or partial cystectomy is the more advisable treatment.Anaplastic lymphoma kinase (ALK) inhibitor can be considered for selected patients.

**Conclusion:**

Inflammatory myofibroblastic tumor of bladder is benign rare tumor.Both diagnosis and management are challenging due to pathology confusion with sarcomas and rarity of tumor. Transurethral resection is choice of management and partial cystectomy is also alternative. Crizotinib, an anaplastic lymphoma kinase (ALK) inhibitor can be considered in selected patients. Though metastasis and recurrence rare post-operative follow up with clinical and cystoscopy is crucial.

## Highlights

• Bladder IMTs are uncommon subtypes of sarcoma arising from mucosal epithelium and mesentery.
• They can occur various organs like eyes, nose, mouth, digestive tract, lungs, and genital and urinary tracts.
• Typically benign, these tumors can occasionally exhibit local aggression and may recur yet its malignant potential is not well established.
• Diagnosis challenging due to its pathology confusion with bladder sarcomas, immunohistochemical analysis may be necessary to differentiate.
• Transurethral resection of tumor is said to be choice of management but partial cystectomy is also alternative.Crizotinib, an anaplastic lymphoma kinase (ALK) inhibitor can be considered for selected patients.
• Here we report a 52 years old lady with recurrent bladder IMT who was treated with transurethral resection of bladder tumour(TURBT).
• Importance of this report is help us to understand nature, prognosis and management of this rare tumor.

## 1 Case presentation

### 1.1 Initial presentation

A 52-year-old woman from Silte, Ethiopia presented to the Outpatient department with a complaint of painless gross hematuria associated with the passage of clots of two weeks duration. She also complained of dysuria, frequency, and urgency, and easy fatigability of the same day's duration. She denied fever, chronic illness, surgical history, smoking, weight loss, and night sweats. She had a good appetite.

Physical examination was unremarkable.

Laboratory investigations were within normal range, including urine culture that didn't show growth over 24 h, but urine analysis revealed hematuria. Abdominal ultrasound showed an intravesical mass measuring 4 × 3 cm, and the other solid organ scans were unremarkable. Chest X-ray showed no signs of metastasis. Cystoscopy showed a big solid broad-based growth over the trigone area. She underwent TURBT; tumor resection was completed in two sessions five days apart, and the specimen was sent for pathology evaluation. The pathology result turned out to be an inflammatory fibroblastic tumor; no malignant cells were seen. Then it was decided to follow the patient. However, in the meantime, the patient disappeared from follow-up.

## 2 Current visit

She presented to the Emergency department with gross painless intermittent hematuria associated with easy fatigability, tinnitus, and vertigo of 2 weeks duration.

## 3 Physical examination

Her ECOG was one. She was an acutely sick-looking woman with vital signs showing tachycardia to a level of 104, and she had pale conjunctiva. Other systems were unremarkable.

She was investigated; her Hemoglobin was 4 mg/dl. Urine analysis showed hematuria. Other blood tests were within normal range, including renal function tests. A 24-h urine culture showed no growth.

**Fig. 1:**
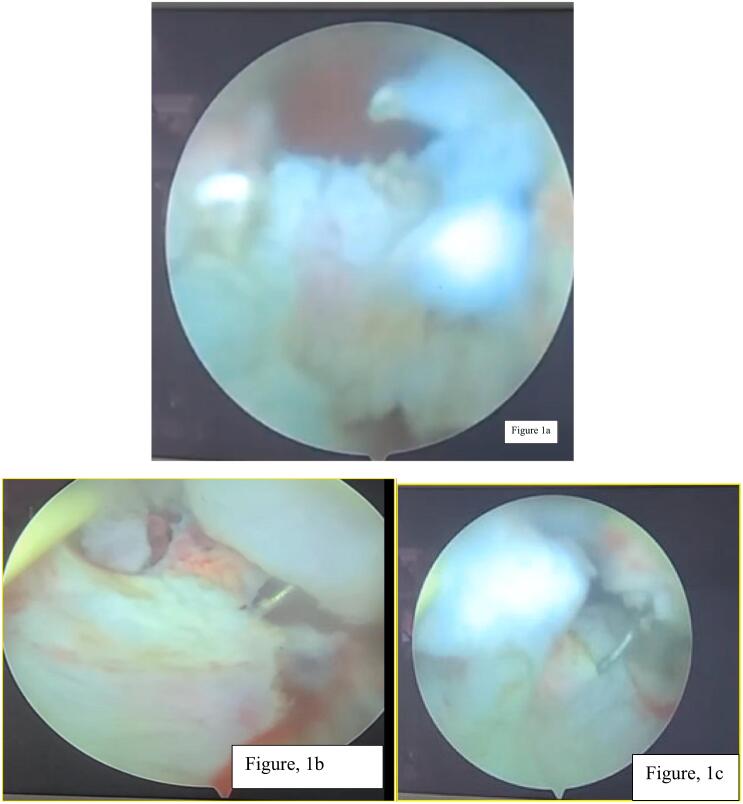

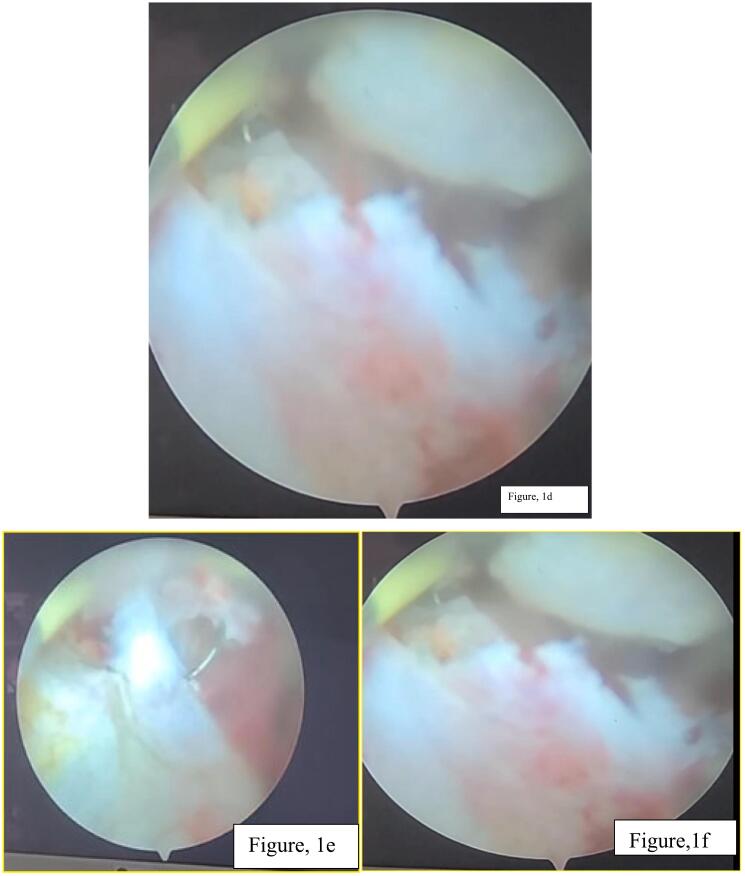
a, b, c, d, e, f; Intraoperative pictures showed dense solid broad based bladder mass located just posterior to right ureteric orifice. Whitish tissue with limited vascularity and minimal bleeding.

**Fig. 2:**
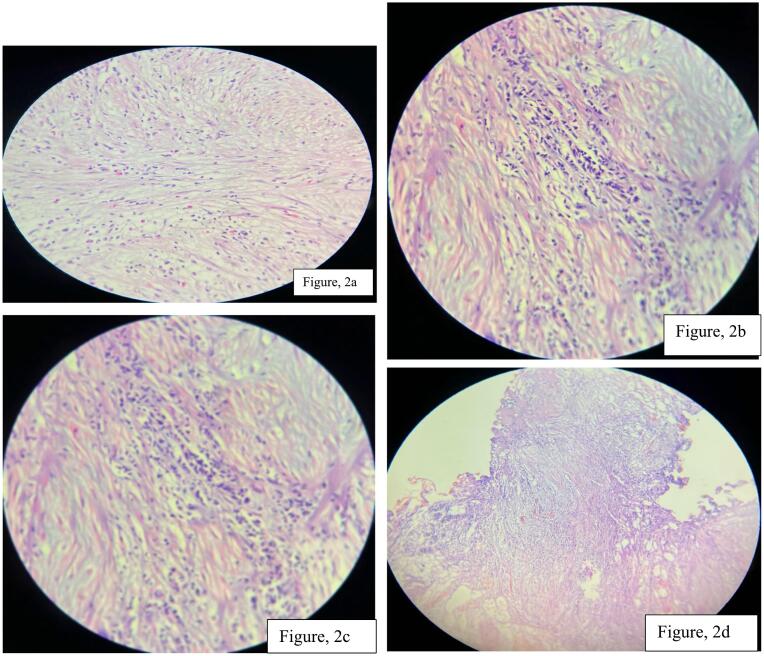
a, b, c, d Histology pictures- showed loose fascicular growth of bland spindle cells with eosinophilia cytoplasm, interspersed by lymphoplasmacytic inflammatory cells in a background of myxoid stroma.

For this, she was transfused with 3 units of packed red blood cells. The patient was optimized and admitted to the ward. The patient was opted, and her management options were briefly discussed, and she and her family preferred transurethral resection. TURBT was done, intra operative finding revealed single solid growth at trigone(Fig. 1a,b,c,d,e,f), it was completed with one session and tissue was sent for pathology evaluation. The biopsy report was consistent with the previous report (Fig. 2a, b,c,d). Immunohistochemistry analysis was not done because the patient couldn't afford it.

## 4 Discussion

Inflammatory myofibroblastic tumor (IMT) is a very rare mesenchymal neoplasm that tends to occur in children and young adults, with a mean age of 9 to 10 years [1].

IMT is of bladder is an uncommon benign tumor of bladder of unknown neoplastic potential characterized by spindle cell proliferation with characteristic fibroinflammatory and pseudosarcomatous appearance. It is also known as pseudosarcoma, atypical myfibroblastic tumor, atypical fibromyxoid tumor, plasma cell granuloma, etc. [2]

It is idiopathic and no known predisposing condition exist for myofibroblastic tumor of the bladder [3]. The most common site for this tumor is lung. It is rare in the genitourinary tract with the most common site being urinary bladder [4].

The first case was reported by Roth in 1980 as unusual pseudosarcomatous lesion of the urinary bladder is described in a thirty-two-year-old woman. The absence of malignant epithelial elements, the benign clinical course, and a history of chronic cystitis support the interpretation of a reactive proliferation [5].

Origin of IMT is controversial, but a recent report suggests that it is neoplastic because of its aggressive behavior, involvement of chromosome 2p23 and cytogenetic clonality. Essential criteria for the diagnosis of IMT are: spindle myoepithelial cell proliferation and lymphocytic infiltrate. Immunohistochemical staining may demonstrate positivity for anaplastic lymphoma kinase, vimentin, cytokeratin. Anaplastic lymphoma kinase (ALK) has been described as a good marker for IMT. Myogenin, a potent marker for rhabdomyosarcoma, helped in exclusion of this tumor [6].

Because of its highly cellular nature and aggressive behavior, it can be confused with malignancy. Initial biopsy and full histopathological examination is recommended where complete resection is problematic.

The clinical presentation of inflammatory myofibroblastic tumors (IMTs) is primarily determined by the tumor's location. In case of bladder IMTs, gross hematuria is the most frequently reported symptom; however, patients may also experience suprapubic pain, lower urinary tract storage symptoms, and in instances of severe hemorrhage, systemic effects related to anemia.

Both the diagnosis because of confusion with malignancy and management due to rarity of IMTs are challenging.

The diagnosis of spindle cell proliferations in the urinary bladder may be very challenging, and occasional cases require adjunctive immunohistochemical studies for classification. When evaluating vesical spindle cell lesions, it is imperative that both the morphologic features and the clinical history be closely correlated because, out of context, the immunophenotypes are relatively nonspecific [12].

Tsuzuki and coworkers examined 16 cases of IMTof the bladder in 14 patients to elucidate the incidence of ALK-1 expression by immunohistochemistry and its diagnostic usefulness.About 71 % of the patients had IMTs of the bladder that were positive for ALK-1 protein, suggesting a neoplastic rather than a reactive process. ALK-1 is a useful marker to help differentiate IMTs from other tumors occurring in the bladder [13].

Management is aimed at complete local resection through transurethral resection or partial cystectomy is the more advisable treatment [7].

Though primarily treated via resection however, in cases of recurrence or unresectable tumors, no standard care exists. This group of patients are typically resistant to standard chemotherapy agents. All patients should undergo molecular sequencing of their tumors for actionable targets such as mutations in *ALK* or *NTRK*, which influences available treatment optionshere is a report with crizotinib, an anaplastic lymphoma kinase (ALK) inhibitor, is only approved for non-small-cell lung cancer with ALK mutation, resulted in complete response [8].

A systematic review of total of 75 case reports on bladder IMTs showed transurethral resection of the bladder tumor (TURBT) (34 %), commonly done procedure followed by complementary partial cystectomy (16 %), or TURBT followed by radical cystectomy (4 %). At a mean follow-up of 14 months, the recurrence and metastasis rates were about 9 % and 4 %, respectively in patients undergoing partial cystectomies [14].

The outcome of these tumors can vary depending on anatomic location, with lung and bladder tumors typically having a more favorable outcome. However, IMTs may locally recur in 25 % of patients with abdominopelvic tumors. Additionally, patients may rarely develop metastatic disease; common sites include the lung, liver, bone, and brain [1].

In our case we managed our patient with complete TURBT resection but tumor recurred within eight months of initial resection. Recurrent IMTs are rare but still can be managed with TURBT.

The patient is currently being followed with clinical evaluations, abdominal ultrasound scans, and cystoscopy. At her first clinic visit, three months post-resection, she had no hematuria, reported feeling well clinically, and cystoscopy revealed no evidence of bladder growth.

Follow up of patients with regular cystoscopy and imaging like CT urography, if indicated, every 3 to 6 months is recommended to pick recurrence early.

The inclusion of intraoperative images and detailed pathology findings can be taken as strength of this case report However, the absence of pre-operative cystoscopy and immunohistochemistry reveals areas for improvement in future studies and would have provided a more comprehensive assessment in the current report.

## Patient (parent's) consent

Written informed consent was obtained from the patient for publication of this case report and accompanying images. The options of management and outcomes were discussed thoroughly with patient and family, they preferred to have TURBT.

A copy of the written consent is available for review by the Editor-in-Chief of this journal on request.

## Ethical approval

Ethical approval was provided by the author's institution.

## Funding

N/A.

## Author contribution

1. Abiy Tadele Alene (MD, Urology Resident): Assisted surgery, diagnosed the patient, Conceptualization, Methodology, Manuscript writing, and Submission and followed the patient.
2. Kumlachew Tilahun Tamir (MD, final year Urology resident): Operated the patient and reviewed the final manuscript.
3. Admasu Melaku,MD(MD, Assistant Professor of urology): consultant surgeon and reviewed the final manuscript
4. Melat Debebe Teka(MD, Assistant Professor pathology): diagnosed histology and reviewed the final manuscript.
5. Endale Demeke Dessalew, MD (Urology resident); reviewed manuscript involved in patient follow up.

## Guarantor

Abiy Tadele Alene.

Melat Debebe Teka.

## Research registration number

N/A.

## Declaration of competing interest

No competing financial interests exist.
